# Terahertz-induced acceleration of massive Dirac electrons in semimetal bismuth

**DOI:** 10.1038/srep15870

**Published:** 2015-11-02

**Authors:** Yasuo Minami, Kotaro Araki, Thang Duy Dao, Tadaaki Nagao, Masahiro Kitajima, Jun Takeda, Ikufumi Katayama

**Affiliations:** 1 Department of Physics, Faculty of Engineering, Yokohama National University, Yokohama 240-8501, Japan; 2 International Center for Materials Nanoarchitectonics, National Institute for Materials Science, Tsukuba 305-0044, Japan; 3 CREST, Japan Science and Technology Agency, Kawaguchi 332-0012, Japan; 4 Department of Condensed Matter Physics, Graduate School of Science, Hokkaido University, Sapporo 060-0810, Japan; 5 LxRay Co., Ltd., Nishinomiya 663-8172, Japan; 6 Department of Applied Physics, National Defense Academy, Yokosuka 239-8686, Japan

## Abstract

Dirac-like electrons in solid state have been of great interest since they exhibit many peculiar physical behaviors analogous to relativistic mechanics. Among them, carriers in graphene and surface states of topological insulators are known to behave as massless Dirac fermions with a conical band structure in the two-dimensional momentum space, whereas electrons in semimetal bismuth (Bi) are expected to behave as massive Dirac-like fermions in the three-dimensional momentum space, whose dynamics is of particular interest in comparison with that of the massless Dirac fermions. Here, we demonstrate that an intense terahertz electric field transient accelerates the massive Dirac-like fermions in Bi from classical Newtonian to the relativistic regime; the electrons are accelerated approaching the effective “speed of light” with the “relativistic” beta *β* = 0.89 along the asymptotic linear band structure. As a result, the effective electron mass is enhanced by a factor of 2.4.

Bi is the heaviest naturally occurring atom with an approximately infinite radiative lifetime^1^. The strong central force caused by the highly charged core in a Bi atom gives rise to the strong spin-orbit coupling^2^. As a result, Bi crystals become a semimetal with a very small electron mass and a Fermi wave-vector with a non-parabolic band structure^3^. This peculiar band structure makes Bi crystal a prototypical material demonstrating various exotic physical properties, such as large diamagnetism^4^, strong Shubnikov-de-Haas effects^5^, and quantum size effects^6^. In spite of these fascinating characteristics, the transport dynamics of the highly accelerated electrons, in which drastic modulation of those exotic properties is expected, has not been revealed to date, partly because transport measurements with direct current could not be available due to the low melting point of Bi crystal (544 K): ohmic heat easily melts the crystal structure of Bi.

The conduction electrons in Bi can be described by the extended Dirac Hamiltonian by applying a ***k***·***p*** perturbation theory around *L* point in the Brillouin zone; it has an asymmetric hyperbolic dispersion around the band bottom with an extremely small electron mass, whereas it approaches a linear band dispersion at high energy region^7^,^8^,^9^. Therefore, Bi is a showcase to examine the electron transportation dynamics from classical Newtonian to the relativistic picture via strong acceleration of electrons. Schematic of the band structure described above is shown in Fig. 1, together with the Brillouin zone of Bi. Here, the “speed of light” for the Dirac-like electrons is defined by the gradient of the asymptotic hyperbolic dispersion of the Dirac-like cone at *L* point.

In the present study, we used a high quality crystalline Bi (001) thin film, evaporated on a Si (111) substrate with 7 × 7 reconstructed surface (see the Specimen Preparation section in Methods). In order to access the electron transportation dynamics of the Dirac-like electrons in Bi, we utilized terahertz (THz) time-domain spectroscopy, which is free from thermal effect. To achieve full acceleration of the Dirac-like electrons in Bi, we used a strong electric field transient with a sub-picosecond pulse duration and a maximum electric field of 130 kV/cm which was generated from a LiNbO_3_ single crystal^10^.

Figure 2a shows the experimentally observed electric field waveforms transmitted through the air, bare Si substrate, and the 40-nm-thick Bi film on Si substrate with maximum electric field intensities of 130 and 13 kV/cm. Because of the THz-induced transient current, the transmitted THz waveform through Bi becomes smaller compared with that through the substrate. Further, as shown by arrows to visualize the magnitude of the transmittance, Bi becomes more transparent for the large maximum THz electric field. In order to examine this behavior, we investigated the THz transmittance as a function of field strength in detail, which is summarized in Fig. 2b. Here, the transmittance is averaged over 0.4–1.1 THz, through Bi film on Si substrate relative to that through the bare Si substrate. As shown by circles in Fig. 2b, the transmittance increases as the THz electric field increases. By applying a conventional Drude model to the experimental results, we expect that either the electron density becomes smaller or the effective mass of the electrons becomes larger with increasing the applied electric field strength. However, the model cannot explain these parameter changes at all, since as it only deals with a linear response of the electrons. Since the transmittance strongly depends on the field strength, the observed phenomenon requires a comprehensive model which covers the whole range of THz field strengths.

To highlight the underlying physics of the electron dynamics in Bi, we assumed an isotropic Dirac Hamiltonian, since Bi possesses a three-fold rotational symmetry around the *c*–axis. The electron dynamics against an electromagnetic field with a polarization 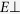 *c*-axis is, therefore, expected to be symmetric, governed by the lowest electron mass of 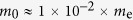 (*m*_e_ is the free electron mass in vacuum) in the binary direction. We also assumed that the electromagnetic response of holes could be negligible, since the effective mass is sufficiently large relative to that of the electrons^11^. The interband excitation is also neglected, since the bandgap at *L* point (40 meV at room temperature) of Bi is considerably larger than the photon energy of the applied THz electric field (2 meV)^12^; the multi-photon absorption process due to 20 photons can be excluded. It is also noteworthy that the observed THz-induced transparency contradicts with the increase of the carrier density due to the multi-photon absorption process.

The equation of motion for the electrons in Bi under an electromagnetic field is


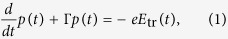


where *p*(*t*) is the kinetic momentum of electrons, Γ is the damping constant, *e* is the elementary charge, and 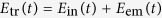 is the electric field at the film transmitted through the specimen. Here, *E*_in_(*t*) and *E*_em_(*t*) are respectively the incident and emitted (or radiated) electric fields from the specimen^13^. Since the thickness of the specimen (*d* = 40 nm) is much smaller than the wavelength of the applied THz field (*λ* ≈ 600 *μ*m), all electrons in the specimen undergo the same driving field *E*_tr_(*t*)^14^. Based on the thin film approximation, *E*_tr_(*t*) is described by the induced current as^15^


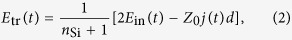


where *Z*_0_ (≈377 Ω) is the impedance of free space and *j*(*t*) = −*nep*(*t*)/m^*^(*t*) is the current density of the Bi film, while *n* is the electron density and m^*^(*t*) is the effective mass. The electron effective mass on the non-parabolic band structure is^16^


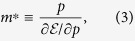


where 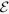 is the energy in the band structure, which can be approximated by a hyperbolic curve (see Fig. 1). Analogous to relativistic mechanics, the energy can be expressed as


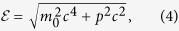


where *m*_0_ is the “rest mass”, and *c* is the “speed of light” in the 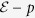 space^17^,^18^. Here, the “rest energy” 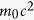 corresponds to 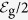, where 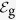 is the bandgap energy. Finally, the equation of motion for Dirac-like electrons in Bi films is given by


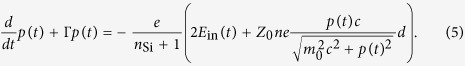


By using these equations under the incident electric field *E*_in_(*t*) with a known quantity without any fitting parameter, the time dependent *p*(t), *j*(*t*), and *E*_tr_(*t*) can be obtained.

Since the second term on the right hand side of Eq. (5) is nonlinear in kinetic momentum, we expect nonlinear dynamics for the Dirac-like electrons in Bi. In particular, the “relativistic” effect can be explicitly observed when sufficiently strong THz electric field is applied. From ref. 19, *c* in Bi is estimated to be 5 × 10^5^ m/s, which is three orders of magnitude smaller than that in vacuum. Therefore, the required field strength for the observation of the relativistic effect is significantly reduced compared with electrons in vacuum; the electric field of 100 kV/cm is enough for the observation. Furthermore, using the above equations, we can evaluate *β* = *v*/*c* to quantify the deviation from the Newtonian mechanics, where *v* is the velocity of electrons defined from Eq. (3) as 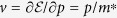.

By using the aforementioned model and the observed electric field waveform transmitted through the air, *E*_in_(*t*), we could calculate the transmitted THz waveforms through Si substrate and the Bi film on it. Details of the calculation are listed in the Calculation section of the Methods. As is shown in Fig. 2a, the calculated THz waveforms reproduce the experimental ones, both in shape and strength, showing that our model can describe the relativistic and nonlinear dynamics of electrons in Bi with no adjustable parameters. Further, the observed increase in THz transmittance is well reproduced by the calculation, as shown by a solid curve in Fig. 2b, indicating that the induced nonlinearity originates from the Dirac-like electrons in Bi. We estimated the maximum electric-field dependence of the effective mass as shown in Fig. 2c. Above ~100 kV/cm, the maximum effective mass increases linearly, as is expected from the denominator of Eq. (3). The effective mass is increased by a factor of 2.4 at the maximum electric field of 130 kV/cm.

Figure 3a–c show the summary of the calculated time evolution of *β*, normalized effective mass, and kinetic energy under the intense electric field transient with a maximum field of 130 kV/cm. The physical parameters in Fig. 3a–c reach their maximum at *t* = 0.8 ps, at which the electric field becomes the strongest. The intense THz field transient significantly modulates *β* from classical Newtonian (*β* = 0) to the relativistic region (*β* = 0.89). Furthermore, the electrons are excited up to 28 meV from the bottom of the band by the intense electric field transient, whose energy is 1.4 times larger than that of 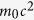 (20 meV). Considering the small photon energy of the THz electric field (2 meV), the total energy given to the electrons via the kinetic momentum is correspondingly high. Figure 3d shows the calculated displacement of electrons in real space. The electron goes up to −260 nm at 1.2 ps, and subsequently, comes back to −80 nm. Note that the final stable position of the electron, after passing through the THz electric field transient, is not zero. The electron effective mass becomes heaviest at *t* = 0.8 ps under the intense THz field, and as a result, the electron becomes hard to move, leading to the shorter trip distance. The −260 to +50 nm displacement is fairly large; however, the flat-homogeneous area of the crystalline Bi film is large enough for this displacement to be valid^20^. We also note that we could not observe any THz-induced transparency in polycrystalline Bi thin films. The use of the high quality Bi film with a large homogeneous area is crucial for observing the electron acceleration with large nonlinearity. Finally, the calculated temporal motion of the electrons in the 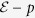 space is summarized in Fig. 3e. The intense THz electric field drives the electrons far above the Fermi energy of the Dirac-like band. As shown in Fig. 3e, this makes the electrons swing back and forth between the classical Newtonian and “relativistic mechanics” along the binary direction at *L* point. Direct measurements of the electron current and speed are our next target for further elucidating the acceleration of the Dirac-like electrons^21^.

In conclusion, we have demonstrated the acceleration of the Dirac-like electrons in high quality single-crystalline Bi thin film using intense THz electric field transient. The electrons were accelerated up to near the *c* with *β* of 0.89 along the almost linear band structure of Bi, in which the “relativistic dynamics” dominates the motion of electrons. As a result, the electron mass is enhanced by a factor of 2.4. We believe that our work opens the door for investigating strong coupling between Dirac-like electrons and electromagnetic radiation, and further, for understanding the solid-state analogue of relativistic mechanics and the massive Dirac dispersion.

## Methods

### Specimen Preparation

We prepared a semimetal Bi thin film (001) with 40-nm-thickness using molecular beam epitaxy (<10^−7^ Pa). The film was deposited on a Si (111)–7 × 7 reconstructed surface^20^, and post-annealed at 350 K to improve the crystallinity. Because of the approximate lattice matching of the Si surface with lattice constant of Bi, we could obtain a highly flat single-crystalline Bi film, whose quality was evaluated by a RHEED measurement and an atomic force microscope (AFM). High-resistivity Si is used as the substrate to avoid a nonlinear effect of the substrate under intense THz field irradiation^22^.

### Calculation

Using Eq. (5) in the main text, we calculated the kinetic momentum of electrons irradiated under an intense THz field transient. The values used in the calculation are 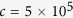 m/s 19, 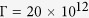 s^−1^ 23, 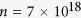 cm^−3^ 23, 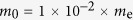 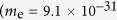 kg)^11^, and 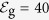 meV at 300 K^12^.

## Additional Information

**How to cite this article**: Minami, Y. *et al.* Terahertz-induced acceleration of massive Dirac electrons in semimetal bismuth. *Sci. Rep.* **5**, 15870; doi: 10.1038/srep15870 (2015).

## Figures and Tables

**Figure 1:**
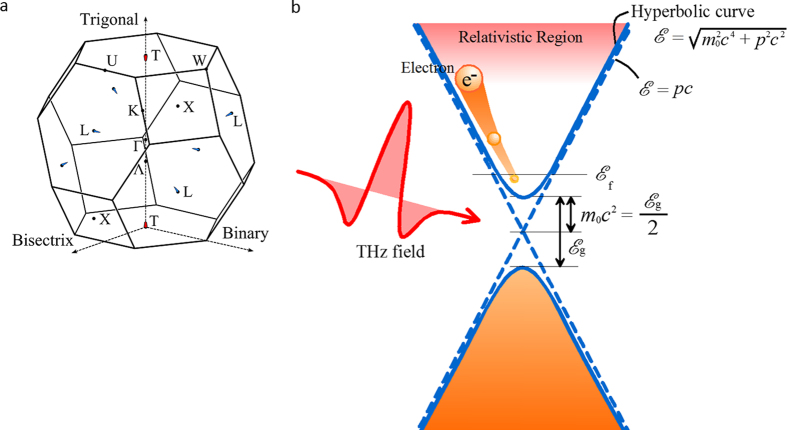
Schematic of the Brillouin zone and the band structure and electron motion around the *L* point in Bi under THz field irradiation. (**a**) Brillouin zone of Bi. (**b**) Electron motion in 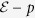 space under an intense THz electric field transient. The intense THz field transient can accelerate the electrons into “relativistic” region. Therefore, the “relativistic” effect must be taken into account in the analysis of the electron motion.

**Figure 2:**
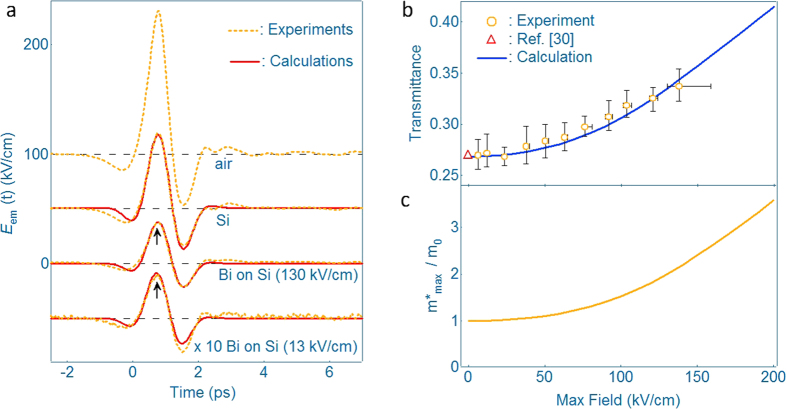
Observed and calculated electric field waveforms, and THz-field-induced transmittances and effective mass. (**a**) Observed electric field waveforms transmitted through the air, bare Si substrate, and the 40-nm-thick Bi film on Si substrate under maximum electric field intensitis of 130 and 13 kV/cm. Calculated electric field waveforms transmitted through the bare Si substrate, and the 40 nm-thick Bi film on Si substrate. Each curve is shown with an appropriate offset. (**b**) Circles and a triangle (ref. 23) show the power transmittances averaged over 0.4–1.1 THz observed at different THz electric field intensities. The transmittance becomes higher with increasing THz electric field. The solid curve shows the calculated power transmittance, which is in good agreement with the values obtained experimentally. (**c**) Normalized effective mass as a function of the maximum electric field. The effective mass increases linearly with increasing the maximum electric field above ~100 kV/cm.

**Figure 3:**
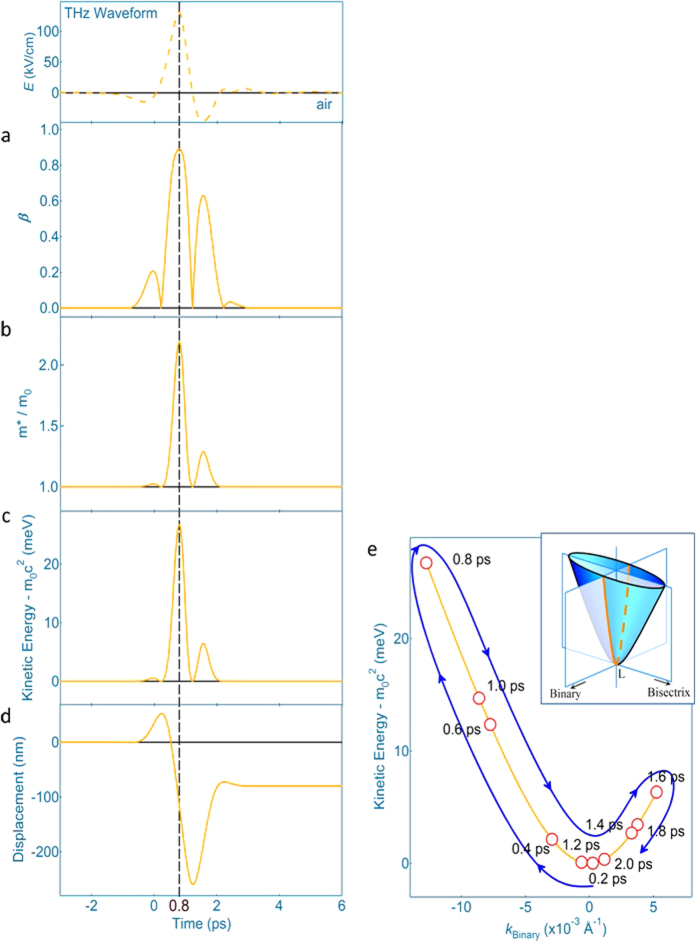
Time evolution of physical parameters representing the “relativistic” dynamics and the THz-field-induced electron trajectory in 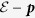 space. (**a**) Temporal dependence of *β*. (**b**) Temporal dependence of the effective mass normalized by the “rest mass”. (**c**) Temporal dependence of the kinetic energy. The electrons are excited up to 28 meV from the bottom of the band. (**d**) THz-field-induced electron motion in real space. The physical values in a–c reach maximum when the applied electric field reaches its maximum (see the upmost figure). (**e**) The electron trajectory in 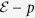 space. The inset shows a picture of the Dirac-like cone at *L* point and the direction of the electron motion.

## References

[b1] De Marcillac P., Coron N., Dambier G., Leblanc J. & Moalic J.-P. Experimental detection of *α*-particles from the radioactive decay of natural bismuth. Nature 422, 876 (2003). 12712201 10.1038/nature01541

[b2] Hirahara T. *et al.* Role of Spin-Orbit Coupling and Hybridization Effects in the Electronic Structure of Ultrathin Bi Films. Phys. Rev. Lett. 97, 146803 (2006). 17155281 10.1103/PhysRevLett.97.146803

[b3] Liu Y. & Allen R. E. Electronic structure of the semimetals Bi and Sb. Phys. Rev. B 52, 1566 (1995). 10.1103/physrevb.52.1566 9981218

[b4] Fukuyama H. & Kubo R. Interband Effects on Magnetic Susceptibility. II. Diamagnetism of Bismuth. J. Phys. Soc. Jpn. 28, 570 (1970).

[b5] Édel’man V. S. Electrons in bismuth. Adv. Phys. 25, 555 (1976).

[b6] Asahi H., Humoto T. & Kawazu A. Quantum size effect in thin bismuth films. Phys. Rev. B 9, 3347 (1974).

[b7] Wolff P. A. Matrix elements and selection rules for the two-band model of bismuth Original Research Article. J. Phys. Chem. Solids 25, 1057 (1964).

[b8] Fuseya Y., Ogata M. & Fukuyama H. Spin-Hall Effect and Diamagnetism of Dirac Electrons. J. Phys. Soc. Jpn. 81, 093704 (2012).

[b9] Zhu Z., Fauqué B., Fuseya Y. & Behnia K. Angle-resolved Landau spectrum of electrons and holes in bismuth. Phys. Rev. B 84, 115137 (2011).

[b10] Hebling J., Almási G., Kozma I. Z. & Kuhl J. Velocity matching by pulse front tilting for large-area THz-pulse generation. Opt. Express 10, 1161 (2002). 19451975 10.1364/oe.10.001161

[b11] Madelung O. in Semiconductors: Data Handbook 3rd Edition (Springer, Berlin, 2003).

[b12] Vecchi M. P. & Dresselhaus M. S. Temperature dependence of the band parameters of bismuth. Phys. Rev. B 10, 771 (1974).

[b13] Kuehn W. *et al.* Coherent Ballistic Motion of Electrons in a Periodic Potential. Phys. Rev. Lett. 104, 146602 (2010). 20481951 10.1103/PhysRevLett.104.146602

[b14] Shih T. *et al.* Nonlinear response of radiatively coupled intersubband transitions of quasi-two-dimensional electrons. Phys. Rev. B 72, 195338 (2005).

[b15] Katayama I. *et al.* Ferroelectric Soft Mode in a SrTiO_3_ Thin Film Impulsively Driven to the Anharmonic Regime Using Intense Picosecond Terahertz Pulses. Phys. Rev. Lett. 108, 097401 (2012). 22463665 10.1103/PhysRevLett.108.097401

[b16] Ariel V. Effective Mass and Energy-Mass Relationship. *arXiv*:1205.3995 (2012).

[b17] Mikhailov S. A. & Ziegler K. New Electromagnetic Mode in Graphene. Phys. Rev. Lett. 99, 016803 (2007). 17678180 10.1103/PhysRevLett.99.016803

[b18] Lax B., Mavroides J. G., Zeiger H. J. & Keyes R. J. Infrared Magnetoreflection in Bismuth. I. High Fields. Phys. Rev. Lett. 5, 241 (1960).

[b19] Hirahara T. *et al.* Quantum well states in ultrathin Bi films: Angle-resolved photoemission spectroscopy and first-principles calculations study. Phys. Rev. B 75, 035422 (2007).

[b20] Nagao T. *et al.* Surface pre-melting and surface flattening of Bi nanofilms on Si(111)–7×7. Surf. Sci. 590, L247 (2005).

[b21] Schiffrin A. *et al.* Optical-field-induced current in dielectrics. Nature 493, 70 (2013). 23222521 10.1038/nature11567

[b22] Wen H., Wiczer M. & Lindenberg A. M. Ultrafast electron cascades in semiconductors driven by intense femtosecond terahertz pulses. Phys. Rev. B 78, 125203 (2008).

[b23] Yokota K. *et al.* Surface Metallic States in Ultrathin Bi(001) Films Studied with Terahertz Time-Domain Spectroscopy. Appl. Phys. Lett. 100, 251605 (2012).

